# Changes in DXA-derived lean mass and MRI-derived cross-sectional area of the thigh are modestly associated

**DOI:** 10.1038/s41598-019-46428-w

**Published:** 2019-07-11

**Authors:** Dallin Tavoian, Kwasi Ampomah, Shinichi Amano, Timothy D. Law, Brian C. Clark

**Affiliations:** 1 Ohio Musculoskeletal and Neurological Institute (OMNI), Ohio University, Athens, OH 45701 USA; 2 Division of Geriatric Medicine, Ohio University, Athens, OH 45701 USA; 3 Department of Family Medicine, Ohio University, Athens, OH 45701 USA; 4 Department of Biomedical Sciences, Ohio University, Athens, OH 45701 USA; 5 Department of Biomedical Engineering, University of Southern California, Los Angeles, CA 90007 USA

**Keywords:** Skeletal muscle, Outcomes research

## Abstract

Dual-energy X-ray absorptiometry (DXA) derived measures of lean mass demonstrate strong associations with magnetic resonance imaging (MRI) derived measures of muscle volume (MV) in cross-sectional studies, however, few studies have compared *changes* in response to an intervention. The purpose of this study was to determine the accuracy of DXA at detecting changes in lean mass, using MRI-derived MV as a reference standard. 10 male and 16 female subjects (29.2 ± 9.5 years) underwent DXA and MRI scans before and after a 10-week resistance training intervention. DXA thigh lean mass was compared to MRI mid-thigh MV, and percent change in size was compared between MRI and DXA. There was a strong correlation between measures cross-sectionally (*r* = 0.89) in agreement with previous investigations. However, there was a modest correlation of percentage change over time between methods (*r* = 0.49). Bland-Altman plots revealed that the amount of random error increased as the magnitude of the change from baseline increased. DXA measures of change in lean mass were modestly associated with MRI measures of change in MV. While there are several advantages to using DXA for the measurement of lean mass, the inability to accurately detect changes over time calls into question its use in clinical trials.

## Introduction

Skeletal muscle plays a critical role in health as it permits the performance of exercise, as well as the activities of daily living. It also plays a central role in whole-body protein metabolism and glucose-regulation^1,2^. Scientists and clinicians have long had an interest in quantifying skeletal muscle tissue mass or size^3,4^, and today the change in skeletal muscle mass or size is the primary outcome in many clinical trials due to its relationship with disease progression and physical function^5–7^. Accordingly, it is crucial that the most appropriate technology for quantifying muscle mass or size be chosen for studies of this nature.

The most accurate methods of quantifying skeletal muscle size *in vivo* are, arguably, magnetic resonance imaging (MRI) and computed tomography (CT), as these technologies have been shown to be near perfectly correlated with cadaveric values (r = 0.99) when full-length limb scans were used^8,9^. Accuracy of MRI single-slice anatomical cross-sectional area (CSA) has also been demonstrated in multiple muscle groups^10,11^. These methods are ideal in terms of accuracy, but the high cost of instrumentation, lack of equipment availability, and the expertise required to operate the equipment and analyze the data often preclude the use of these devices in many research settings, particularly late-stage, large-scale clinical trials^12^. As such, dual-energy X-ray absorptiometry (DXA) has gained popularity over the years through its ease of use, reduced cost, and accessibility. While MRI and CT measures are estimates of muscle volume (MV) or CSA, depending on the variable of interest, DXA measures fat mass, bone mineral content, and lean mass, the last including connective tissue, water and organs^13,14^. For simplicity, these estimates will, at times, be collectively referred to as “muscle size” in this article.

A multitude of skeletal muscle size comparative studies have been conducted between DXA and MRI, as well as between DXA and CT, in a variety of populations^15–19^. These comparative studies reported strong associations (correlation coefficient values ranging from 0.86–0.97), for both whole-body and regional scans^15–19^. However, these above-mentioned studies were based on cross-sectional designs, and less is known about the agreement between various indices of change in skeletal muscle size over time.

Only a few studies have directly compared longitudinal changes in skeletal muscle size using both DXA and MRI/CT^16,19–23^. These studies reported strong associations between temporally matched measures (e.g., MRI- and DXA-derived absolute measures at baseline)^16,19–21^. However, when comparing percent-change over time, these studies have indicated discrepant findings with a few studies suggesting much lower associations (explained variances [R^2^-values] on the order of 4–33%)^19–21^, while others suggested higher levels of concordance (61% and 77%)^16,23^. The lack of understanding about how well various assessment methods of skeletal muscle size agree in longitudinal studies is a serious problem as it relates to the design and interpretation of clinical trials. For instance, in one study it was reported that 3-months of resistance exercise training in adolescents resulted in a significant increase in appendicular skeletal muscle size when MRI was used as the quantification method, but no significant difference in lean mass with DXA^20^. Another study, this time in older adults, reported increases in thigh muscle size after a 1-year resistance training protocol when measured with CT, but not DXA^22^. These findings are similar to the recently reported results of a clinical trial that investigated the effectiveness of a myostatin-inhibitor for enhancing skeletal muscle size in older adults^24^. In this study, the primary outcome was total body lean mass assessed *via* DXA, while a secondary outcome was thigh skeletal muscle size assessed *via* MRI. The data were discrepant, with the DXA-derived estimate indicating no significant increase, whereas the MRI-derived estimate demonstrated an increase. While these measures are not always directly comparable, results of this nature highlight the need to better understand how DXA- and MRI-derived measures of change in skeletal muscle mass and size compare, as this knowledge has implications for interpreting findings as well as the design of clinical trials. This is particularly alarming when one considers that DXA is, by far, the most common method to assess skeletal muscle mass/size in randomized control trials evaluating muscle mass/size^25^, and DXA has recently been recommended as the “reference standard” for the measurement of muscle mass^26^. Accordingly, the purpose of this study was to examine the relationship between MRI- and DXA-derived measures of thigh skeletal muscle size/mass in a cross-sectional analysis as well as a longitudinal analysis where resistance exercise served as a stimulus for skeletal muscle adaptation.

## Results

**Figure 1:**
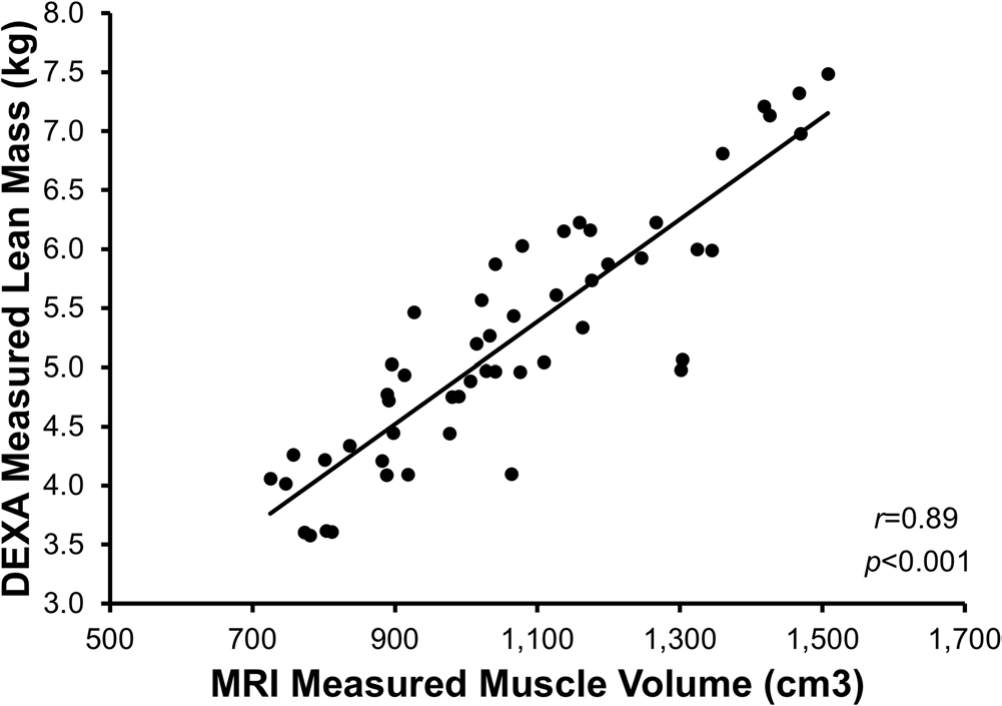
Correlation between MRI and DXA measures cross-sectionally. A strong positive association was observed between whole-thigh MRI- and DXA-derived measures at baseline (*r* = 0.89, p < 0.001).

**Figure 2:**
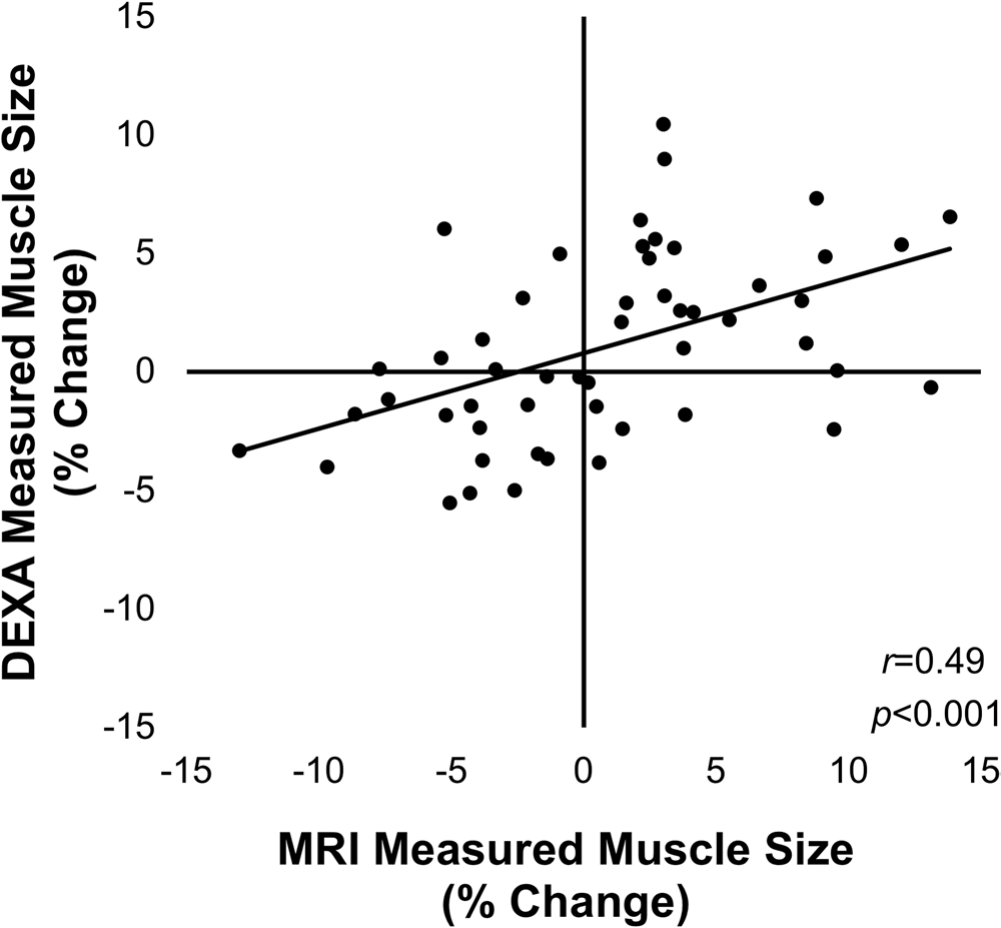
Correlation between MRI and DXA measures of percent change. There was only a modest positive association between the percentage change in estimates of muscle size between the MRI and DXA techniques, such that explained variance was only 24% (*r* = 0.49 p < 0.01).

**Figure 3:**
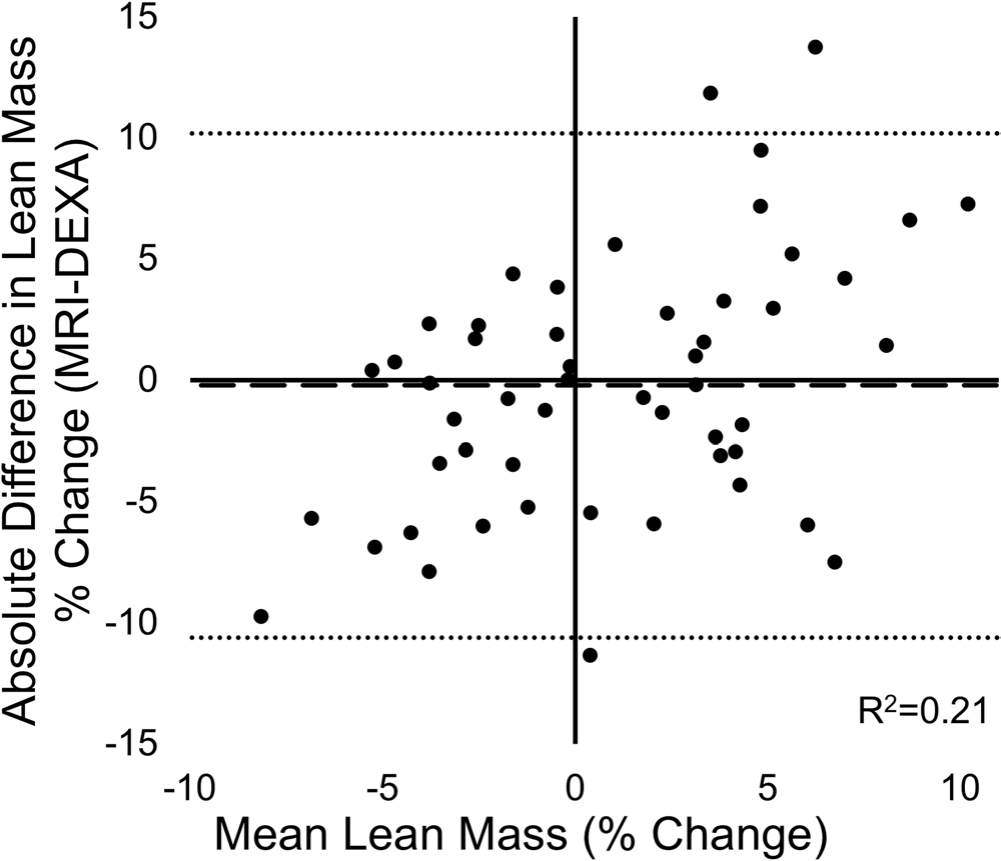
Bland-Altman plot of agreement between MRI- and DXA-derived measures of percent change in muscle size. There was a relationship between the error (difference between MRI and DXA) and the mean percent change in skeletal muscle size, indicating heteroscedacity. Upper LOA was 10.19% and lower LOA was −10.61% (dotted lines). DXA demonstrated a bias of −0.21% (dashed line).

There was a strong positive correlation between whole-thigh MRI- and DXA-derived measures at baseline (*r* = 0.89, p < 0.001; Fig. 1) and after the 10-week exercise program (*r* = 0.90, p < 0.001). However, there was only modest positive correlation of the percentage change calculated over time between the two respective techniques (*r* = 0.49 p < 0.001; Fig. 2). Bland-Altman plots revealed that there was a relationship between the error (i.e., the difference between the two techniques) and the mean percent change in skeletal muscle size, signifying heteroscedasticity (Fig. 3). The ratio LOA was 10.40, indicating that differences due to measurement error should be no more than 10.40% in either direction in 95% of cases. Examination of individual cases demonstrated that only 46% of scans agreed within 3%. For those differing in their estimate by >3%, we noted that in 43% of cases the MRI resulted in higher estimate of percent change, whereas in 57% of cases the DXA resulted in a higher estimate of percent change. Further, for those differing in their estimate by >3%, five cases displayed hypertrophy via MRI but atrophy via DXA, while seven cases displayed hypertrophy via DXA but atrophy via MRI.

When comparing DXA percent change against MRI percent change, DXA displayed an average error of 4.16%, and overestimated muscle mass in 52% of cases. Average error of overestimations was 4.21% with a maximum difference from MRI of 11.32%, while average error of underestimations was 4.11% with a maximum difference from MRI of 13.77%. This resulted in a negligible bias of 0.21% in the negative direction.

## Discussion

The purpose of this study was to examine the relationship between MRI- and DXA-derived measures of thigh MV and lean mass, respectively, in a cross-sectional analysis as well as a longitudinal analysis where resistance exercise served as a stimulus for skeletal muscle adaptation. Our study demonstrates a strong association between MRI- and DXA-derived values when compared at a single time point, in agreement with previous cross-sectional validation studies comparing MRI and DXA measurements of appendicular muscle size. However, DXA-derived measures of percent change in lean mass were modestly associated with MRI-derived measures of MV, and less than half agreed within 3%. These findings demonstrate the imprecision of DXA as a tool to measure changes in appendicular muscle size in response to exercise. Muscle size and change in muscle size have been synonymous terms in clinical trial research, at least in relation to measurement strategies and recommendations. Very recently DXA was recommended as the “reference standard” for measuring lean mass based on the premise that there is a strong association between cross-sectional DXA and MRI or CT measurements^26^. These authors further recommended DXA as a tool to monitor changes in lean mass. Our findings strongly question the validity of this recommendation, at least as it relates to quantifying changes in muscle mass over time (e.g., active muscle wasting).

While cross-sectional validation comparisons are common, there have been a limited number of investigations that directly compared DXA-derived changes in lean mass against MRI or CT over time, with conflicting results. Nindl *et al*.^23^ reported a strong correlation (*r* = 0.88) between single-slice MRI and DXA measures of percent change in muscle size in adult women after a 6-month exercise program. However, in this study the lower leg and foot were included in the DXA regional analysis, and whole-leg muscle mass was directly compared to a MRI-derived mid-thigh single-slice CSA. Bridge *et al*.^16^ also reported a strong association (*r* = 0.78) between MRI- and DXA-derived percent change in muscle size, but with a more equivalent analysis. They compared MRI scans of the middle one-third of the thigh against a DXA-customized region of interest (ROI) of the middle one-third of the thigh in children over a three-year period. It is of note that these changes were not in response to an exercise intervention, but rather due to the normal maturation process. Further, these children exhibited increases in muscle size in the range of 50–100% over the three-year follow-up period^16^. In contrast, we report no significant intervention-related increase in muscle size *per se* using either DXA or MRI data; however, there was a large degree of heterogeneity in response to the intervention with some individuals exhibiting MRI-derived increases as high as ~13% and others exhibiting decreases on the order of ~7% with only modest agreement between measures (r = 0.49). Therefore, it appears that DXA may be able to detect very large changes in lean mass (i.e. > 50%), though it should be noted that differences between the two measures in the previous study were still quite large despite the high level of agreement, with thigh mass increasing 55.7% from baseline as measured by DXA but 73.5% as measured by MRI^16^. Additionally, changes of this magnitude are limited to unique scenarios (e.g. adolescent development), and DXA has been unable to accurately detect smaller changes in lean mass. For instance, a three-month resistance training program in obese adolescents resulted in significant increases in leg muscle size as measured by MRI, but not DXA^20^. Investigators reported a modest association between whole-body MRI and DXA measures of change in muscle/lean mass (*r* = 0.57), but a weak association between appendicular (leg) measures of change in muscle/lean mass (*r* = 0.20)^20^.

In recent years there has been a growing interest in the aging field to quantify muscle mass for the diagnosis of sarcopenia^27,28^, as well as evaluation of the effectiveness of exercise, nutrition and pharmaceutical interventions to enhance muscle mass in older adults to prevent or treat sarcopenia^29,30^. Within the older adult population several studies have compared changes in muscle using various imaging modalities. Specifically, Hansen *et al*.^19^ and Delmonico *et al*.^21^ compared changes in muscle size/mass in older adults after surgical repair of the femoral neck and in response to resistance training, respectively. CT- and DXA-derived percent change values were only modestly associated in both studies (*r* = 0.51 and 0.53), and Delmonico and colleagues reported a limits of agreement (LOA) ratio of 7.2%. These findings, combined with our own results, demonstrate the lack of sensitivity in detecting small to modest changes in muscle size inherent in DXA. The previous discrepancy in reported agreement between DXA and MRI can be largely explained by the discordance of anatomical regions (e.g. whole-leg mass vs. mid-thigh 10 mm single-slice CSA) assessed by the two measures, as well as the magnitude of reported change.

There are several possible reasons for the differences seen between cross-sectional and longitudinal correlations of muscle mass measurements between DXA and MRI. One source of inaccuracy likely stems from the compounding error of multiple DXA measurements. The relatively small error inherent in a DXA cross-sectional measurement of muscle size can be compounded when multiple measures are taken and converted to percent-change in size. Another source of inaccuracy relates to the assumptions of DXA technology. Error seen in response to same-day repeated measures is attributed exclusively to machine error and rater error^31^, however, one cannot assume that this holds true after an intervention, as exercise training can induce tissue changes that may impact X-ray attenuation^23^. The component profile for lean soft tissue mass includes proteins, glycogen, soft tissue minerals, and water, and altering the relative composition of these components through an intervention (i.e., exercise or weight loss) changes the attenuation coefficient, potentially violating the assumptions necessary for the accurate use of DXA technology^32^. For example, weight loss can induce fluid retention, and DXA may misinterpret the relatively greater hydrated lean soft tissue mass as a higher-density tissue^33^. It is likely that further changes in the relative composition of the components of lean mass occur in response to different interventions, but additional work is needed to develop a greater understanding of the variables that can affect DXA measures over time.

As mentioned earlier, there was a heterogeneous response to the resistance training in our study, resulting in muscle hypertrophy in some participants, as would be expected, but atrophy in others. We believe this is due to several reasons. First, the exercise interventions were not overall strenuous in terms of the intensity/load as well as the total volume. This is particularly the case for the low-load resistance training control group. Second, the muscle groups utilized during exercise training were somewhat different than the muscle groups analyzed herein. One of the benefits of using the MRI is the ability to isolate individual muscles or muscle groups for analysis. In our study participants performed knee extension exercises, but not knee flexion exercises. Using the MRI, we would be able to analyze changes in the quadriceps muscle group, excluding all other muscles/tissues. However, the DXA is unable to separate muscle groups and can only quantify the mass of transverse sections of the body (i.e., total lean soft tissue mass from hip to knee). With this in mind, we chose to analyze all muscles of the thigh from the MRI scans to make our analysis more comparable to the DXA scans. Including all thigh muscles in the MRI analysis when only the knee extensors were targeted likely resulted in a misleading interpretation of the effect of the chosen exercise on muscle hypertrophy. However, the intention of this manuscript is to determine the level of agreement between two measurement techniques, and not necessarily to determine if a certain exercise protocol resulted in muscle hypertrophy. Therefore, we believe the heterogeneity of the sample strengthens our findings, as detecting decreases in muscle size is equally important to detecting increases, at least in sarcopenic populations.

This does raise the question of how large of a change in muscle size is considered clinically relevant. Roth *et al*.^34^, reported that MRI-derived thigh muscle volume increased on average 4–6% in older adults and 4–8% in younger adults after six months of resistance training performed three times per week. Additionally, in a meta-analysis Isodori *et al*.^35^, reported a 2.7% increase in fat free mass in response to testosterone treatment. While statistically significant, it remains unclear how large of an increase should be considered clinically meaningful^36^. A full discussion on the clinical relevance of muscle size and changes in muscle size is outside the scope of this manuscript, however, based on past reports we believe that a change in muscle size of 3% is at least experimentally relevant. Therefore, tools to measure change in muscle size should be highly accurate to detect such small changes.

Our findings, when considered in light of the extant literature, raise questions around prior studies and trials using DXA to detect changes in muscle mass. This is exemplified in previous studies demonstrating changes in muscle size when measured by MRI or CT, but not by DXA^20,22,24^. In light of our findings and similar observations from others^19–21^, we recommend that future studies give strong consideration to their selection of modalities for assessing muscle mass. While DXA may be a cost- and time-effective method to assess muscle mass cross-sectionally, a more in-depth investigation into the accuracy of DXA at detecting changes in muscle mass should be performed prior to its widespread acceptance as the “reference standard”.

### Limitations

In the present study the full-thigh DXA lean mass was compared against five-slice MRI-derived MV taken from the middle third of the thigh. It should be noted that middle third of the quadriceps is where most hypertrophy occurs, compared with proximal and distal regions^37^, and we limited our analysis to that region. To determine whether differences in the ROI influenced our findings we created a customized ROI to analyze the middle third of the thigh using the DXA software, making MRI and DXA scans directly comparable. We found that there was even less agreement between the two measures when using the customized ROI (see Supplementary Table S1). As such, we do not believe that the differences in the ROI drove the findings of a weak association between the two techniques for assessing percent change in muscle size.

Another potential limitation is the field strength of the magnet used in the current study. Our 0.25-tesla magnet will produce lower quality scans than a stronger-tesla magnet, ultimately reducing the accuracy of scan analysis. However, this potential limitation was compensated for by increasing the scan time to reduce the signal-to-noise ratio^38^.

## Conclusion

The purpose of this study was to examine the relationship between MRI- and DXA-derived measures of thigh skeletal muscle size in a cross-sectional analysis as well as a longitudinal analysis where resistance exercise served as a stimulus for skeletal muscle adaptation. The measurement of lean mass in the thigh region via DXA was highly correlated to MRI-derived MV values, as has been seen in previous cross-sectional studies. However, DXA was unable to accurately detect changes in lean mass in response to resistance exercise. These findings suggest that future studies must give strong consideration to their selection of modalities for assessing lean mass. Further, these findings strongly question the recent suggestion that DXA be considered the “reference standard” for measuring muscle.

## Methodology

### Study participants & exercise program

Twenty-six adults (29.2 ± 9.5 years, 16 females and 10 males) participated in this study. The data for this manuscript was derived from a previously described randomized control trial comparing two different exercise programs for effectiveness in inducing skeletal muscle adaptations^39,40^. See Table 1 for participant characteristics. Study participants were randomly assigned to one of two exercise intervention groups, and a whole-body DXA scan (thigh region used for analysis) and a mid-thigh MRI were obtained before and after the 10-week exercise program. The use of human subjects and all experimental procedures were approved by the Ohio University Institutional Review Board committee and written informed consent was obtained from each participant.

**Table 1:** Participant Characteristics.

		BFR	Control	*p*-values
	Participants (N)	12	14	
	% Female	75	50	
	Age (yrs)	28.0 ± 9.9	30.1 ± 9.4	0.58
	Body Mass (kg)	72.7 ± 15.6	72.9 ± 14.1	0.97
	Height (cm)	168.2 ± 9.4	171.3 ± 9.1	0.40
	BMI	25.6 ± 5.1	24.6 ± 3.2	0.57
*MRI*	Baseline Thigh Volume (cm^3^)	988.5 ± 171.5	1124.8 ± 224.6	0.02*
*MRI*	Post Thigh Volume (cm^3^)	999.6 ± 160.4	1125.4 ± 225.0	0.03*
*MRI*	Thigh Volume Change (%)	1.49 ± 5.83	0.28 ± 6.18	0.48
*DXA*	Baseline Thigh Lean Mass (kg)	4.98 ± 0.81	5.43 ± 1.15	0.11
*DXA*	Post Thigh Lean Mass (kg)	4.98 ± 0.80	5.53 ± 1.18	0.06
*DXA*	Thigh Lean Mass Change (%)	0.20 ± 3.98	1.79 ± 3.71	0.14

BFR, blood flow restricted experimental group; BMI, body mass index; DXA, dual-energy X-ray absorptiometer; MRI, magnetic resonance imager; *significant difference between BFR and control group.

Study participants were randomly assigned to either a low-load blood flow restricted resistance training group (n = 12) or a low-load resistance training control group (n = 14). Supervised exercise training sessions were conducted twice per week for 10 weeks, with exercise intensity set at 25% of each of the participant’s maximal voluntary isometric strength (MVIC). MVIC was obtained at baseline and reassessed during the fifth week of the training schedule. Participants performed three sets of leg extensions, calf raises, and arm curls to failure with 60 seconds rest between sets. The BFR group had pressure applied to the proximal limbs by a KAATSU Master device (KAATSU Training Japan Co., Ltd., Tokyo, Japan) until circulation was impeded, but not occluded. For full details on the exercise program and other trial design parameters please see our in depth description of the protocol/trial^39,40^.

### Assessment of skeletal muscle size

#### Magnetic resonance imaging

**Figure 4:**
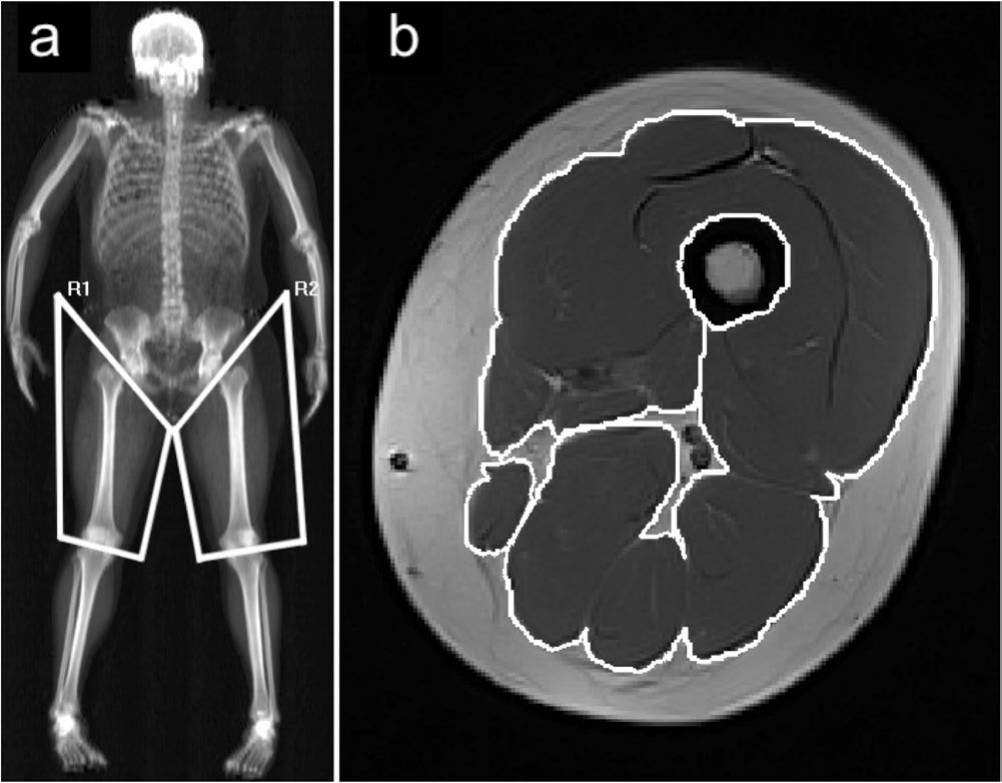
DXA and MRI regions used for analysis. (**a**) Diagram showing regions of interest in DXA scans. (**b**) Magnetic resonance image of the mid-thigh, muscles included for analysis traced in white.

MRI scans were performed with a 0.25-Tesla Musculoskeletal MRI system (Esaote G-Scan Brio, Genoa, Italy) to acquire contiguous transverse T-1 weighted spin echo image slices in the thigh region with a slice thickness of 10 mm and an inter-slice distance of 10 mm. To ensure consistency among subjects, the isocenter was positioned at mid-thigh, midway between the patella and the inguinal crease, and the subjects were supine. Images were transferred to a computer for calculation of whole-thigh muscle anatomical CSA. Beginning with the slide with the first discernable visual of the rectus femoris and including the subsequent four proximal slides, total thigh muscle area was traced using a polygon tool, excluding bone, as well as fat tissue surrounding the muscles (MIPAV version 7.3.0) (Fig. 4). Intramuscular fat was then subtracted by applying a shading correction to each slide, determining average voxel density and standard deviation voxel density from a sample of the lightest area of fat tissue, computing a cutoff value at three standard deviations darker than the sample voxel density, and excluding all pixels with a voxel density at or below the computed value. Pre- and post-intervention slides were displayed simultaneously, and slides were visually compared to ensure that tracing patterns were identical and that the same structures were excluded (i.e. neurovascular bundle, intermuscular fat) on both slides before CSA values were recorded. This process was completed for each analyzed slide, resulting in five measures of whole-thigh CSA with intermuscular and intramuscular fat excluded for both pre and post time points. Muscle volume was calculated using the Cavalieri method [MV = T (A_1_ + A_2_ + A_3_ + A_4_ + A_5_)], where MV = muscle volume, T = the known distance between slices, and A = area^41^.

#### Dual-energy X-Ray absorptiometry

DXA scans (Hologic Discovery QDR model Series, Waltham, MA, USA) were performed to assess thigh lean mass immediately after the MRI was performed, and at least three days after the last exercise session. A whole-body scan was performed, and whole-body and regional lean tissue mass was determined using the system’s software package (Hologic APEX, Version 4.0.2). The thigh region was defined and isolated for analysis using a method similar to those reported previously (Fig. 4)^42,43^. Participants were scanned at the same time of day pre- and post-intervention and were encouraged to maintain a similar sleeping and eating schedule for both scans. Subjects were advised to report to the laboratory in a hydrated state and they were given scrubs to wear during the scan. They were also given the opportunity to use the restroom prior to the scan. Care was taken to follow The International Society for Clinical Densitometry guidelines for positioning during the scan^44^.

#### Repeatability of MRI and DXA measures

All scans were analyzed by the same investigator blinded to the experimental group of the subjects. Repeated measurement coefficient of variation (CV) was calculated for the investigator based on repeated measures of randomly selected MRI and DXA scans from 10 subjects on different days separated by at least two weeks. The MRI method had a CV of 1.3%, while the DXA method had a CV of 0.96%. It should be noted that this method only assessed measurement variability of the investigator and not machine variability, as the same scan was analyzed twice at different time points. True CV is expected to be higher if machine variability were to be included.

### Statistical analysis

The statistics software SPSS version 24.0 (SPSS Incorporated, Chicago IL) was used for statistical analysis. Pearson correlations were calculated to determine strength of association between MRI and DXA measures at baseline and after the 10-week exercise program. Percent-change from baseline was then calculated for MRI and DXA measures for each participant (e.g., [MRI_post_ − MRI_pre_]/MRI_pre_ * 100), and a Pearson correlation was calculated between percent-change MRI and percent-change DXA values. For the purpose of our analyses, left and right legs were considered independent of one another, and as such there were 52 data points for comparison.

We utilized the LOA method (a measure of absolute reliability), which is a statistical technique that is useful in partitioning out systematic bias vs. random error^45,46^. In doing this, Bland–Altman plots were generated for each variable and analyzed for the presence of heteroscedasticity (heteroscedasticity: residuals are not equally distributed; homoscedasticity: residuals are approximately equal). This was determined by examining the association (R^2^) between the absolute differences and the mean values. R^2^ values between 0 and 0.1 were considered homoscedastic (no relation between error and the size of the measured variable)^46^. R^2^ values greater than 0.1 were heteroscedastic (amount of random error increases as the measured values increases) and the LOA ratio were then calculated^46^. The LOA ratio is calculated using the following equation: LOA ratio = [(SD_diffs_/AVG_means_) 1.96] 100. Where SD_diffs_ is the standard deviation of all of the difference scores (%Δ MRI − %Δ DXA calculated for each subject), AVG_means_ is the average of all of the mean scores (mean of %Δ MRI and %Δ DXA for each subject), and the factor of 1.96 represents the inclusion of 95% of observations of the difference score. The LOA ratio is interpreted as “any two tests will differ due to measurement error by no more than X% either in the positive or negative direction”^46^.

### Research involving human subjects

All procedures performed in studies involving human participants were in accordance with the ethical standards of the institutional and/or national research committee and with the 1964 Helsinki declaration and its later amendments or comparable ethical standards.

### Informed consent

Informed consent was obtained from all individual participants included in the study.

## Supplementary information

Dataset 1

## Data Availability

All data is available in Supplementary Table S1.
