# Supplementary material for: Changes in DXA-derived lean mass and MRI-derived cross-sectional area of the thigh are modestly associated
Source: Sci Rep. 2019 Jul 11;9:10028. doi: 10.1038/s41598-019-46428-w (PMC6624257; doi:10.1038/s41598-019-46428-w)
Supplement: Supplementary file 1 — Dataset 1 [file 41598_2019_46428_MOESM1_ESM.docx]

Changes in DXA-derived lean mass and MRI-derived cross-sectional area of the thigh are modestly associated

Dallin Tavoian, Kwasi Ampomah, Shinichi Amano, Timothy D. Law, and Brian C. Clark

**Table S1. Baseline, post-intervention, and percent change values for MRI, DXA, and DXA customized region of interest measurements of the thigh**

|  |  |  | **MRI (cm^3^)** | | |  | **DXA (kg)** | | |  | **DXA ROI (kg)** | | |
| --- | --- | --- | --- | --- | --- | --- | --- | --- | --- | --- | --- | --- | --- |
| **Subject** | **Group** | **Leg** | **Pre** | **Post** | **% Change** |  | **Pre** | **Post** | **% Change** |  | **Pre** | **Post** | **% Change** |
| 1 | Con | Left | 1021.31 | 1114.28 | 9.10 |  | 5.57 | 5.84 | 4.87 |  | 1.88 | 1.87 | -0.34 |
|  |  | Right | 1040.70 | 1177.16 | 13.11 |  | 5.87 | 5.83 | -0.66 |  | 1.92 | 1.88 | -1.98 |
| 2 | BFR | Left | 894.98 | 980.54 | 9.56 |  | 5.03 | 5.03 | 0.06 |  | 1.20 | 1.24 | 2.97 |
|  |  | Right | 926.38 | 1013.77 | 9.43 |  | 5.46 | 5.33 | -2.43 |  | 1.35 | 1.33 | -1.51 |
| 3 | BFR | Left | 746.34 | 835.85 | 11.99 |  | 4.02 | 4.23 | 5.37 |  | 1.28 | 1.37 | 7.36 |
|  |  | Right | 724.81 | 825.01 | 13.83 |  | 4.06 | 4.33 | 6.55 |  | 1.35 | 1.36 | 0.47 |
| 4 | BFR | Left | 1136.88 | 1091.97 | -3.95 |  | 6.15 | 6.01 | -2.36 |  | 1.75 | 1.76 | 0.66 |
|  |  | Right | 1078.32 | 1021.95 | -5.23 |  | 6.03 | 5.92 | -1.81 |  | 1.69 | 1.71 | 0.74 |
| 5 | Con | Left | 1417.80 | 1312.83 | -7.40 |  | 7.21 | 7.13 | -1.15 |  | 2.27 | 2.26 | -0.26 |
|  |  | Right | 1507.65 | 1377.20 | -8.65 |  | 7.48 | 7.35 | -1.78 |  | 2.41 | 2.38 | -0.99 |
| 6 | Con | Left | 1005.62 | 1010.34 | 0.47 |  | 4.88 | 4.81 | -1.44 |  | 1.47 | 1.44 | -1.72 |
|  |  | Right | 1027.98 | 1013.77 | -1.38 |  | 4.97 | 4.79 | -3.66 |  | 1.46 | 1.35 | -7.15 |
| 7 | Con | Left | 800.99 | 818.77 | 2.22 |  | 4.22 | 4.44 | 5.31 |  | 1.23 | 1.18 | -4.43 |
|  |  | Right | 757.06 | 769.09 | 1.59 |  | 4.26 | 4.38 | 2.92 |  | 1.27 | 1.33 | 4.59 |
| 8 | BFR | Left | 836.09 | 803.93 | -3.85 |  | 4.34 | 4.40 | 1.37 |  | 1.44 | 1.51 | 4.92 |
|  |  | Right | 890.80 | 921.21 | 3.41 |  | 4.72 | 4.97 | 5.25 |  | 1.58 | 1.65 | 4.02 |
| 9 | Con | Left | 1032.80 | 1064.26 | 3.05 |  | 5.27 | 5.74 | 9.00 |  | 1.43 | 1.59 | 11.35 |
|  |  | Right | 1014.20 | 1044.55 | 2.99 |  | 5.20 | 5.74 | 10.46 |  | 1.47 | 1.53 | 4.05 |
| 10 | Con | Left | 803.16 | 847.16 | 5.48 |  | 3.62 | 3.70 | 2.21 |  | 1.01 | 1.05 | 3.56 |
|  |  | Right | 772.76 | 800.78 | 3.63 |  | 3.60 | 3.70 | 2.60 |  | 1.06 | 1.07 | 0.37 |
| 11 | BFR | Left | 780.75 | 810.13 | 3.76 |  | 3.58 | 3.62 | 1.00 |  | 1.09 | 1.12 | 2.66 |
|  |  | Right | 810.97 | 830.97 | 2.47 |  | 3.61 | 3.78 | 4.80 |  | 1.07 | 1.07 | 0.19 |
| 12 | BFR | Left | 897.55 | 910.60 | 1.45 |  | 4.45 | 4.34 | -2.40 |  | 1.32 | 1.26 | -4.56 |
|  |  | Right | 980.07 | 985.66 | 0.57 |  | 4.75 | 4.57 | -3.83 |  | 1.44 | 1.42 | -1.58 |
| 13 | Con | Left | 1359.73 | 1473.93 | 8.40 |  | 6.81 | 6.89 | 1.21 |  | 2.07 | 2.09 | 0.81 |
|  |  | Right | 1426.15 | 1469.54 | 3.04 |  | 7.13 | 7.36 | 3.21 |  | 2.19 | 2.18 | -0.29 |
| 14 | BFR | Left | 888.88 | 850.89 | -4.27 |  | 4.77 | 4.71 | -1.42 |  | 1.43 | 1.40 | -1.85 |
|  |  | Right | 913.24 | 873.83 | -4.31 |  | 4.94 | 4.69 | -5.08 |  | 1.47 | 1.40 | -4.88 |
| 15 | BFR | Left | 888.15 | 843.05 | -5.08 |  | 4.09 | 3.87 | -5.50 |  | 1.20 | 1.11 | -7.21 |
|  |  | Right | 881.47 | 862.69 | -2.13 |  | 4.21 | 4.15 | -1.38 |  | 1.21 | 1.18 | -2.36 |
| 16 | Con | Left | 1301.06 | 1200.42 | -7.74 |  | 4.98 | 4.99 | 0.14 |  | 1.67 | 1.65 | -1.20 |
|  |  | Right | 1303.53 | 1233.13 | -5.40 |  | 5.07 | 5.10 | 0.60 |  | 1.69 | 1.62 | -4.24 |
| 17 | BFR | Left | 1163.16 | 1142.94 | -1.74 |  | 5.34 | 5.15 | -3.46 |  | 1.81 | 1.77 | -2.22 |
|  |  | Right | 1108.63 | 1071.50 | -3.35 |  | 5.04 | 5.05 | 0.11 |  | 1.77 | 1.82 | 2.79 |
| 18 | Con | Left | 1469.41 | 1490.12 | 1.41 |  | 6.98 | 7.13 | 2.12 |  | 2.33 | 2.33 | -0.27 |
|  |  | Right | 1467.54 | 1596.54 | 8.79 |  | 7.32 | 7.86 | 7.35 |  | 2.31 | 2.54 | 9.81 |
| 19 | Con | Left | 1075.63 | 935.52 | -13.03 |  | 4.96 | 4.80 | -3.31 |  | 1.32 | 1.32 | -0.08 |
|  |  | Right | 1040.74 | 939.81 | -9.70 |  | 4.97 | 4.77 | -4.02 |  | 1.34 | 1.27 | -4.84 |
| 20 | Con | Left | 1158.98 | 1156.88 | -0.18 |  | 6.23 | 6.21 | -0.21 |  | 1.97 | 1.98 | 0.75 |
|  |  | Right | 1174.06 | 1146.81 | -2.32 |  | 6.16 | 6.36 | 3.12 |  | 1.99 | 2.12 | 6.56 |
| 21 | Con | Left | 917.79 | 952.92 | 3.83 |  | 4.09 | 4.02 | -1.79 |  | 1.24 | 1.35 | 8.83 |
|  |  | Right | 1063.20 | 1107.00 | 4.12 |  | 4.10 | 4.20 | 2.53 |  | 1.31 | 1.40 | 7.32 |
| 22 | BFR | Left | 1176.09 | 1201.05 | 2.12 |  | 5.74 | 6.10 | 6.41 |  | 1.89 | 1.98 | 4.96 |
|  |  | Right | 1198.35 | 1230.48 | 2.68 |  | 5.87 | 6.20 | 5.61 |  | 1.94 | 2.02 | 4.38 |
| 23 | BFR | Left | 1344.41 | 1292.84 | -3.84 |  | 5.99 | 5.76 | -3.73 |  | 2.11 | 2.02 | -4.10 |
|  |  | Right | 1266.29 | 1232.98 | -2.63 |  | 6.23 | 5.92 | -4.97 |  | 2.20 | 2.07 | -5.73 |
| 24 | Con | Left | 1324.07 | 1326.05 | 0.15 |  | 6.00 | 5.97 | -0.44 |  | 1.78 | 1.82 | 2.44 |
|  |  | Right | 1245.83 | 1228.26 | -1.41 |  | 5.93 | 5.92 | -0.18 |  | 1.74 | 1.75 | 0.49 |
| 25 | BFR | Left | 1125.82 | 1218.50 | 8.23 |  | 5.61 | 5.78 | 3.01 |  | 1.81 | 1.87 | 3.17 |
|  |  | Right | 1066.36 | 1137.01 | 6.62 |  | 5.44 | 5.63 | 3.65 |  | 1.63 | 1.76 | 7.71 |
| 26 | Con | Left | 976.65 | 925.08 | -5.28 |  | 4.44 | 4.71 | 6.04 |  | 1.42 | 1.39 | -1.70 |
|  |  | Right | 989.31 | 980.32 | -0.91 |  | 4.76 | 4.99 | 4.99 |  | 1.42 | 1.51 | 6.07 |

MRI, DXA, and DXA customized ROI values for all subjects at baseline (Pre) and after 10 weeks of low-load resistance exercise (Post). BFR, blood flow restriction group; Con, control group; DXA, dual-energy X-ray absorptiometry; MRI, magnetic resonance imaging; ROI, DXA customized region of interest equivalent to MRI region of interest
